# A *Lobularia maritima* LmSAP protein modulates gibberellic acid homeostasis via its A20 domain under abiotic stress conditions

**DOI:** 10.1371/journal.pone.0233420

**Published:** 2020-05-19

**Authors:** Rania Ben Saad, Walid Ben Romdhane, Wafa Mihoubi, Anis Ben Hsouna, Faical Brini

**Affiliations:** 1 Biotechnology and Plant Improvement Laboratory, Centre of Biotechnology of Sfax, University of Sfax, Sfax, Tunisia; 2 Plant Production Department, College of Food and Agricultural Sciences, King Saud University, Riyadh, Saudi Arabia; 3 Department of Life Sciences, Faculty of Sciences of Gafsa, Gafsa, Tunisia; Institute of Genetics and Developmental Biology Chinese Academy of Sciences, CHINA

## Abstract

Stress-associated proteins (SAPs) are favorable targets to improve stress tolerance in plants, owing to their roles in developmental processes and stress responses. However, the role of SAPs and the molecular mechanisms by which they regulate plant stress responses remain poorly understood. Previously, it was reported that *LmSAP* expression was upregulated by various abiotic stressors in *Lobularia maritima*, and that transgenic tobacco lines with constitutively expressed *LmSAPΔA20* and *LmSAPΔA20-ΔAN1* showed dwarf phenotypes due to the deficiency of cell elongation under salt and osmotic stresses. In this study, we examined the function of A20 domain in the GA pathway in response to abiotic stresses. Transient expression of acGFP-LmSAPΔA20 and acGFP-LmSAPΔA20-ΔAN1 in onion epidermal cells demonstrated that these fused proteins were localized in the nucleo–cytoplasm. However, the truncated form acGFP-LmSAPΔAN1 was localized in the nucleus. Moreover, comparison of native and truncated LmSAP showed dramatic structural changes caused by the deletion of the A20 domain, leading to loss of function and localization. Interestingly, overexpression LmSAP and truncated LmSAPΔAN1 led to up-regulation of GA biosynthetic genes and increased total gibberellins (GAs) content, corresponding with accelerated development in transgenic tobacco plants. Moreover, the dwarf phenotype of the transgenic lines that express *LmSAPΔA20* and *LmSAPΔA20-ΔAN1* under stress conditions was fully restored by the application of exogenous GA_3_. These findings improve our understanding of the role of LmSAP in regulating GA homeostasis, which is important for regulating plant development under abiotic stress conditions.

## Introduction

Plants are sessile organisms throughout their life cycle, and their ability to cope with adverse conditions (biotic and abiotic) through a variety of physio-biochemical processes and various stress-related signaling pathways is crucial for survival [1, 2]. Perception of stressful environmental conditions induces a specific stress hormone signature specifying a proper response, which is efficient in terms of fitness. Several plant growth regulators, such as phytohormones, regulate many physiological and metabolic processes that help plants to adapt to environmental stresses [3, 4]. The phytohormone gibberellin (GA), also known as gibberellic acid [5], regulates various crucial growth and developmental processes, such as seed germination, stem elongation, leaf expansion, and reproductive development, in most plant species [6]. Among the different GAs identified from plants, a few acts as endogenous plant growth regulators (bioactive GAs), including GA1, GA3, GA4, and GA7, but most are precursors [6]. *Arabidopsis* mutants with defect in GA biosynthesis or signaling show severe dwarfism and delayed flowering [7, 8]. Dwarfism is a common phenotype for mutants defective in GA-synthesis enzymes such as the 2-oxoglutarate–dependent dioxygenases GA20-oxidase (GA20ox) and GA3-oxidase (GA3ox) [9]. However, overexpression of *GA2ox1* or *GA2ox3* in transgenic rice can inhibit stem elongation [10, 11]. Emerging evidence shows that GA signaling is involved in abiotic stress. In the case of GA signaling, GA responses are repressed by DELLA proteins (named for a conserved DELLA amino acid motif). SCF^SLY/GID2^ (Skp1, Cullin, and F-box complex) directly interacts with DELLAs, leading to ubiquitination and degradation of the DELLA repressor, thus promoting GA-mediated transcription [12, 13]. Under abiotic stresses, the accumulation of DELLA proteins could partly restrain plant growth, suggesting that through the modulation of GA levels plants can control their growth and resistance to abiotic stress [14, 15].

The stress-associated protein (SAP) family is characterized by the presence of A20/AN1 zinc-finger domains and is highly conserved in all plant species [16]. *Arabidopsis* contains 14 *SAP* genes, while rice, maize, and *Populus* were found to have 18, 11, and 19 genes, respectively. Expression of several *SAP* genes from each of these species is responsive to multiple stresses, and transgenic plants that over-express various SAPs show improved stress tolerance phenotypes [16]. Recently, it has been revealed that ZFP185, an A20/AN1-type zinc-finger protein (ZPF), participates in the regulation of growth and stress responses in rice by modeling GA and abscisic acid (ABA) biosynthesis [17]. Liu et al. [18] demonstrated that *OsDOG*, an A20/AN1 ZFP gene, plays a novel role in GA homeostasis and cell elongation by regulating the expression of genes involved in GA metabolism in rice, indicating the involvement of A20/AN1-type zinc finger proteins in gibberellin (GA) biosynthesis.

In this study, we determined the three-dimensional structure of each form of LmSAP, and the subcellular localization of its truncated constructs. We also examined the involvement of the LmSAP functional domain A20 in the abiotic stress response through modulation of the gibberellic acid (GA) biosynthetic pathway. We report that the expression level of the GA biosynthetic genes *GA3ox*, *GA2ox*, and *GA20ox* was up-regulated and the total endogenous gibberellins (GAs) content was increased in transgenic tobacco plants overexpressing *LmSAP* and *LmSAPΔAN1*. Moreover, overexpression of *LmSAPΔA20* and *LmSAPΔA20ΔAN1* in tobacco plants caused a dwarf phenotype under abiotic stress conditions, which was rescuable by application of exogenous GA3. These results provide novel insight into the role of the A20 domain in regulating GA homeostasis, and underline its importance during periods of abiotic stress.

## Materials and methods

### Plant material, growth condition and hormone treatment

Non-transgenic tobacco (*Nicotiana tabacum* L. cv *Xanthi*) and eight homozygous transgenic tobacco lines expressing *LmSAP*- FL and *LmSAP*-truncated forms used in this study were previously characterized by Ben Saad et al. [19].

Seeds of *L*. *maritima* were harvested in Borj Cedria, a locality close to the Mediterranean seashore, 20 km north of Tunis and planted in plastic pots filled with washed river sand and watered with Milli-Q water until germination, followed by irrigation with 10% Hoagland's nutrient solution for 4 weeks [20]. The pots were placed in a greenhouse under controlled conditions (16 h photoperiod; 300 μmol m^−2^.s^−1^ PAR). The mean temperature and relative humidity were 30 ± 5°C and, 55 ± 5% during the day, and 16 ± 2°C and, 90 ± 5% during the night, respectively. Giberellic acid (GA_3_) was applied by spraying the aerial part of the plants grown in the greenhouse with 5, 10, and 50 μM aqueous solutions containing 0.1% Tween-80 (Sigma-Aldrich). Control plants were sprayed with the same solution. The fully expanded leaves were sampled after 6, 12, 24 and 48 h, frozen in liquid nitrogen, and stored at −80°C for RNA extraction.

### Real-time PCR analysis

Total RNA was isolated from *L*. *maritima* plants treated with GA_3_ using the TRizol reagent (Invitrogen, Carlsbad, CA, USA). RNA was treated with DNase I (MBI; Fermentas, USA) at 37°C for 15 min. To generate the first strand cDNA, DNase-treated RNA samples (5 μg) were reverse-transcribed using M-MLV reverse transcriptase (Invitrogen, Carlsbad, CA, USA). The real-time qPCR was performed in optical 96-well plates on the Roche Light Cycler 480 instrument using SYBR Green I dye (Roche), according to the supplier's manuals. The PCR cycling conditions as were previously described by Ben Saad et al. [19]. Primer pairs were designed using Primer 3 software to ensure gene specificity in the amplification of the *LmSAP* gene and house-keeping Ubiquitin 10 mRNA (*UBQ10*: At4g05320) gene. The sequence of primer pairs is presented in S1 Table. The relative expression level of genes was calculated from triplicate measurements [(CT, _*LmSAP*_-CT, _*UBQ10*_) stressed − (CT, _*LmSAP*_ -CT, _*UBQ10*_) control] [21]. Relative expression ratios of three independent experiments (three biological repetitions) are reported.

NT plants and transgenic lines (A1, A3, B5, B8, C2, C6, D2, and D9) expressing full-length or truncated LmSAP were grown on MS medium (control) and MS medium supplemented with 300 mM NaCl or 300 mM mannitol (stress conditions). After two weeks, leaves were harvested and used for RNA extraction with TRIzol reagent to synthesize the first-strand cDNA using M-MLV reverse transcriptase (Invitrogen, Carlsbad, CA, USA). The transcription levels of *NtGA3ox* (NM_001324779), *NtGA2ox* (NM_001325580), and *NtGA20ox* (XM_016635192) were measured using RT-qPCR as described above, and the specificity of primers was monitored using the melt curve analysis. The primers ACT-F and ACT-R were used to amplify a fragment of the *Actin* gene (380 bp) as the control for equal cDNA loading. All the primers are shown in S1 Table. The experiment was repeated three times to ensure reproducibility.

### Quantitative analysis of the total endogenous gibberellins content

Expanding tobacco plant leaves (NT, A1, A3, B5, B8, C2, C6, D2, and D9) under control and stress conditions were harvested for use in the total gibberellins (GAs) assays. The GAs were quantified according to the protocol described by Fos et al. [22] with some modifications. Freeze-dried tobacco leaves (0.5 g, fresh weight) were homogenized in a cold 100% acetonitrile extraction medium with 30 g/mL of sodium diethyldithiocarbamate as an antioxidant. The homogenate was then subjected to further extraction in the dark at 4°C for 16 h. These extracts were centrifuged at 5,000 *g* (4°C) for 10 min, and the supernatant was dried under low pressure and re-dissolved in 8.0 mL of 0.4 M phosphate buffer (pH 8.0). Trichloromethane (4.0 mL) was added to remove pigment, and 150 mg polyvinylpolypyrrolidone (PVPP) was used to remove the hydroxybenzene. The aqueous phase was subject to extraction using two ethyl acetate treatments. The ethyl acetate phase was dried under low pressure and re-dissolved in 1.0 mL of mobile phase buffer. Finally, the solution was filtered through a 0.45-μm membrane, and the GAs content was analyzed using high-performance liquid chromatography (HPLC) according to the method described by Ben Hsouna et al. [23] with some modifications. An aliquot (15 μL) of the filtered samples was injected into a Waters Symmetry C18 column (4.6 mm x 150 mm, 5 μm) with isocratic elution at a flow rate of 0.8 mL/min at 25°C, using methanol and double distilled water (0.5% acetic acid) as the mobile phase (45:55). The detection of the total GAs was performed at 254 nm using an SPD-10Avp detector (Shimadzu) and co-chromatography with authentic standards (Sigma). The retention times and wavelengths of the characteristic absorbances of the hormones were determined according to authentic standard compounds, while the samples were identified by comparing their elution peaks to these of authentic standard compounds. The total GAs contents in the extracts were quantified using calibration curves. All the analyses were carried out using three biological replicates.

### Exogenous GA_3_ rescue assay

Different salt and osmotic stress assays were conducted with *LmSAP* transgenic tobacco lines (C2, C6, D2, and D9) from the homozygous T3 generation. To assess the growth rate under conditions of salt and osmotic stress with GA_3_ treatment, the seeds of non-transgenic (NT) and *LmSAP* transgenic lines (C2, C6, D2, and D9) were surface sterilized and directly placed on MS medium for 10 days. Thereafter, the seedlings were transplanted into square plates containing MS medium supplemented with 300 mM NaCl+100 μM GA_3_ or 300 mM mannitol+100 μM GA_3_, and incubated in a growth chamber at 22°C under a 16 h light/8 h dark photoperiod. The plates were placed vertically. After a 2-week growth period, the seedlings were measured for growth parameters, and their tissues collected for RT-qPCR analysis. The total leaf areas of *LmSAP* transgenic and NT tobacco seedlings subjected and not subjected to salt and osmotic stress conditions were calculated using the UTHSCA image tool program (http://compdent.uthscsa.edu/dig/itdesc.html). The assays were conducted in triplicates on independent seed lots.

### Subcellular localization

Three LmSAP deletion constructs (LmSAPΔA20, LmSAPΔAN1, and LmSAPΔA20-ΔAN1) were previously generated and cloned into pGEMT-Easy vector [19]. For the evaluation of subcellular localization, the coding regions of the LmSAPΔA20, LmSAPΔAN1, and LmSAPΔA20-ΔAN1 constructs were amplified by PCR using the LmSAP-*BamH*I and LmSAP-*Xba*I primers containing *BamH*I and *Xba*I restriction sites (S1 Table). The amplified fragments were cloned into the *BamH*I/*Xba*I sites of the pCAMBIA2300-acGFP binary vector to generate the following fusion genes driven by the CaMV-35S promoter: acGFP::*LmSAPΔA20*, acGFP::*LmSAPΔAN1*, and acGFP::*LmSAPΔA20-ΔAN* (Fig 3A). The resulting constructs were separately bombarded into onion epidermal cells using the PDS-1000/He gene gun at 1100 psi as described previously by Mare et al. [24], and then cultured in MS medium in the dark at 28°C for 24h. The fluorescence of the GFP fusion proteins was excited at 488 nm with an argon laser and observed with an emission window set at 505–530 nm on the Zeiss LSM confocal laser scanning microscope 510.

### Prediction of the three-dimensional structures of truncated and full-length LmSAP proteins

The amino acid sequences of full-length LmSAP (NCBI accession number: AUN86611), LmSAP-A20, LmSAP-AN1, and LmSAP-A20-AN1 in FASTA format were used as input data into Phyre2 web portal (http://www.sbg.bio.ic.ac.uk/~phyre2/html; [25]) using the intensive modeling mode to predict their tertiary protein structures. The PyMol software (Version 2.3.4; [26]) was utilized to analyze and compare the predicted three-dimensional structures of the truncated proteins. Superimposition of the predicted structures and calculation of the root-mean-square deviation (RMSD) were performed using the SuperPose web server (http://superpose.wishartlab.com; [27]).

### Evaluation of *LmSAP* transgenic tobacco tolerance to salt and drought stresses

To monitor the drought and salt effects under greenhouse conditions, we selected one homozygous transgenic line from each constructs (A1, B5, C2, and D2) and NT plants, previously produced [19]. After germination on MS medium for one month, the seedlings were transferred to pots filled with a 3:1 mixture of soil and peat and then grown in a greenhouse for more than two weeks before being exposed to stress treatments. For normal growth conditions, the transgenic and NT plants were irrigated with water to maintain the relative water content (RWC) of the soil at 75%. For the salt stress treatment, the same irrigation program was used with the addition of 250 mM NaCl in the water until the end of the plant cycle. This NaCl concentration was increased gradually from 50 to 250 mM within the first 20 days of stress treatment. To induce drought stress, the RWC of the soil was maintained at 25%. For all treatments, the plant height, and plant and seed weight (g per plant) were determined.

### Statistical analysis

The data are provided for all experiments as the mean ± standard deviation (S.D.) of three replicates. Statistical analyses were performed using the XLSTAT software with one-way ANOVA [28]. According to Bonferroni’s post-hoc test, the mean values marked with different letters on the figures indicate significant differences at *P* < 0.05.

## Results

### *LmSAP*-truncated domain overexpression in tobacco plants alters growth and seed production under abiotic stress conditions

**Fig 1:**
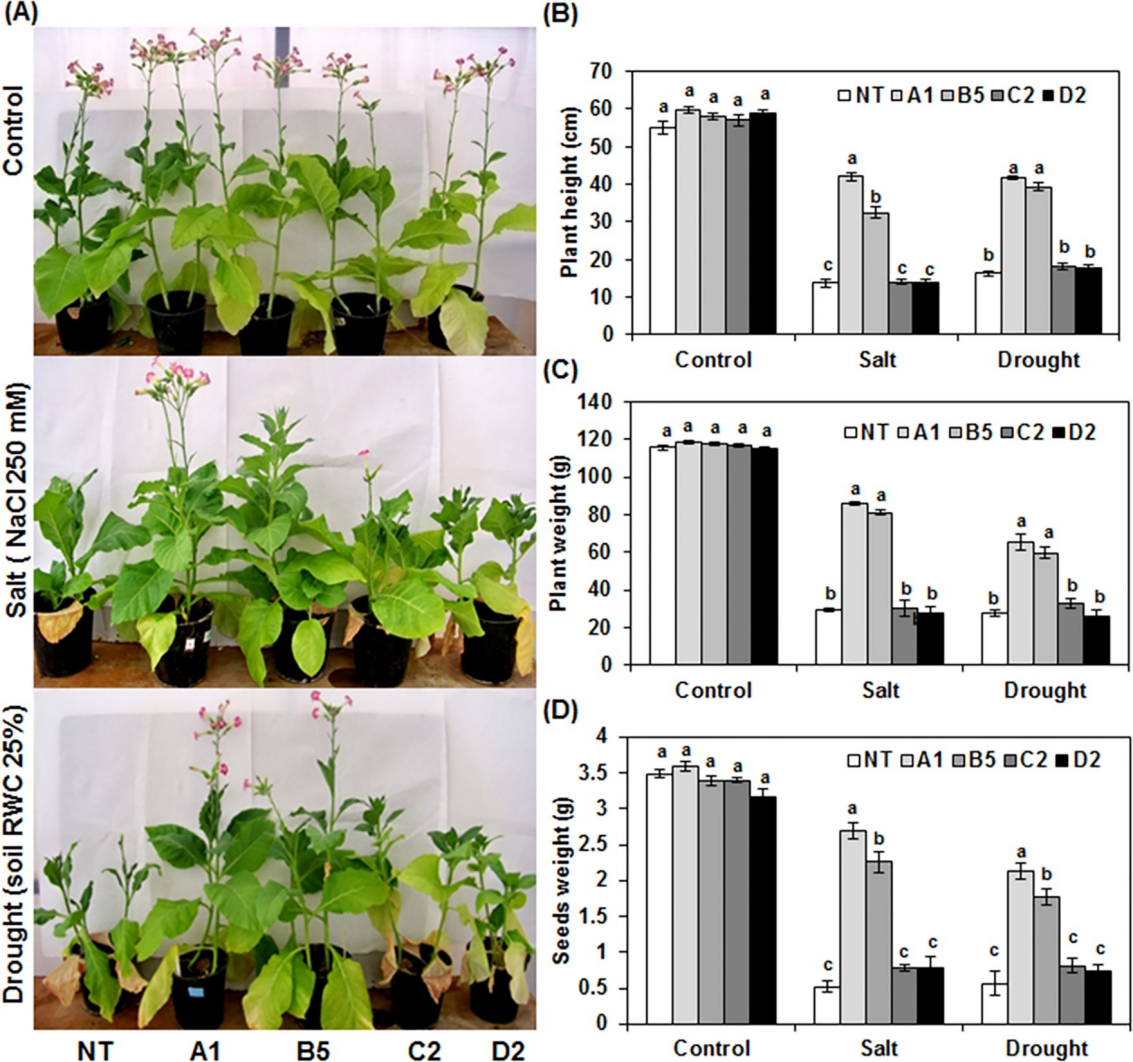
Analysis of salt and drought tolerance of non-transgenic (NT) and *LmSAP-FL*, *LmSAPΔA20*, *LmSAPΔAN1*, and *LmSAPΔA20-ΔAN1* transgenic tobacco plants (named as A1, B5, C2, and D2, respectively) under greenhouse conditions. (**A**) Phenotypes of NT and transgenic plants grown under normal, salt stress (250 mM NaCl), or drought stress (25% RWC in the soil) conditions until the end of the growth cycle. (**B**) Height of NT and transgenic plants transformed with different constructs measured at end of the growth cycle, (**C**) Plant and (**D**) seed weight (g per plant). Values are means ± SE (n = 3). Means denoted by the same letter did not differ significantly at p < 0.05.

Previously, *in vitro* assessment of transgenic tobacco plants overexpressing *LmSAP-FL* (A1 and A3 lines) and *LmSAPΔAN1* (B5 and B8 lines) constructs provided the first evidence of an enhanced tolerance to salt and osmotic stresses when compared with NT and transgenic lines transformed with *LmSAPΔA20* (C2 and C6 lines) and *LmSAPΔA20-ΔAN1* (D2 and D9 lines) constructs [19]. For this purpose, we investigated the effect of *LmSAP* gene (A1 line) and transgenic truncated LmSAP constructs (B5, C2, and D2 lines) on growth and seed production under control conditions, drought stress (soil RWC was maintained at 25%), or salt stress (irrigation with 250 mM NaCl) until the end of the plant cycle in the greenhouse. As shown in Fig 1, ectopic *LmSAP* expression did not affect overall plant morphology as any obvious growth or yield was observed between NT and all *LmSAP* transgenic lines grown under control growth conditions. However, the performance of A1 and B5 transgenic plants was clearly improved under salt and drought stresses when compared with NT, C2, and D2 lines. Indeed, A1 and B5 transgenic lines could continue their growth, reach the flowering stage, and display seeds setting while NT, C2, and D2 lines showed a stunted phenotype (Fig 1A). Similarly, plant height and weight were significantly higher in transgenic plants A1 and B5 than in NT, C2, and D2 lines under both drought and salt stresses (Fig 1B and 1C). Interestingly, the most striking difference between transgenic lines A1 and B5, and NT, C2 and D2 plants was observed in the context of seed production. The A1 and B5 transgenic lines produced two-fold more seeds than did NT, C2 and D2 plants under salt and drought stresses (Fig 1D). All these findings provide evidence that A20 domain of LmSAP protein is essential in enhancing the tolerance to salt and drought stresses in tobacco plants under greenhouse condition.

### *LmSAP* transcription responds to GA_3_

**Fig 2:**
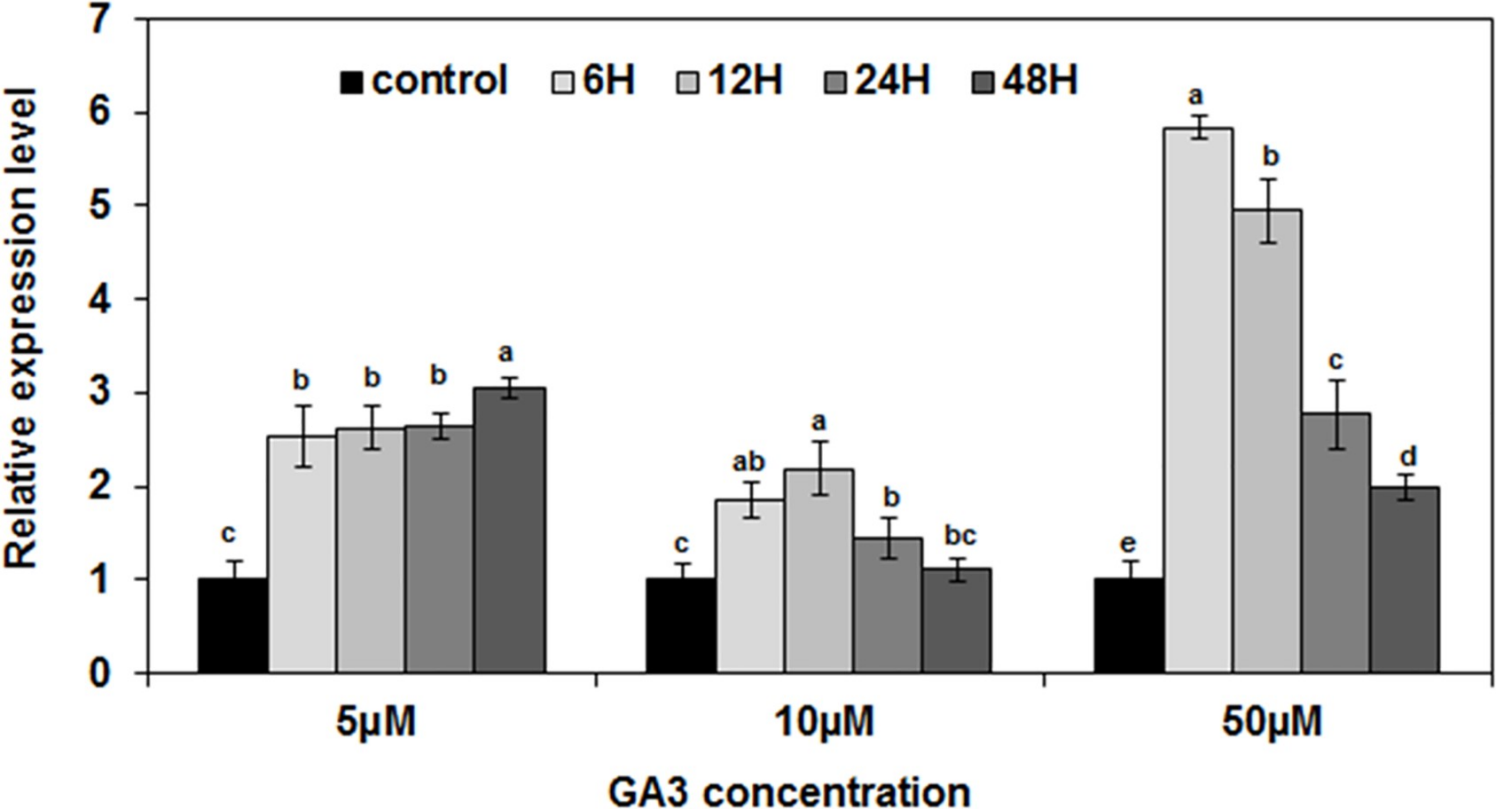
RT-qPCR analysis of the mRNA levels of *LmSAP* in *L*. *maritime* plants treated with 5, 10, or 50 μM of GA3 for 0, 6, 12, 24 and 48 h. The data from RT-qPCR experiments were analysed by the 2^-ΔΔCT^ method and are represented as means ± SE. Means denoted by the same letter were not significantly different at p < 0.05.

We further confirmed the effect of 5, 10 and 50 μM GA_3_ on the accumulation of *LmSAP* transcripts in leaves of *L*. *maritime* using real-time PCR. Our results showed that the mRNA levels of *LmSAP* gene showed highest increase with 50 μM GA_3_ treatment at 12 and 24h after treatment compared with the others GA_3_ concentration tested (Fig 2).

### Sub-cellular localization of three LmSAP-truncated domains

**Fig 3:**
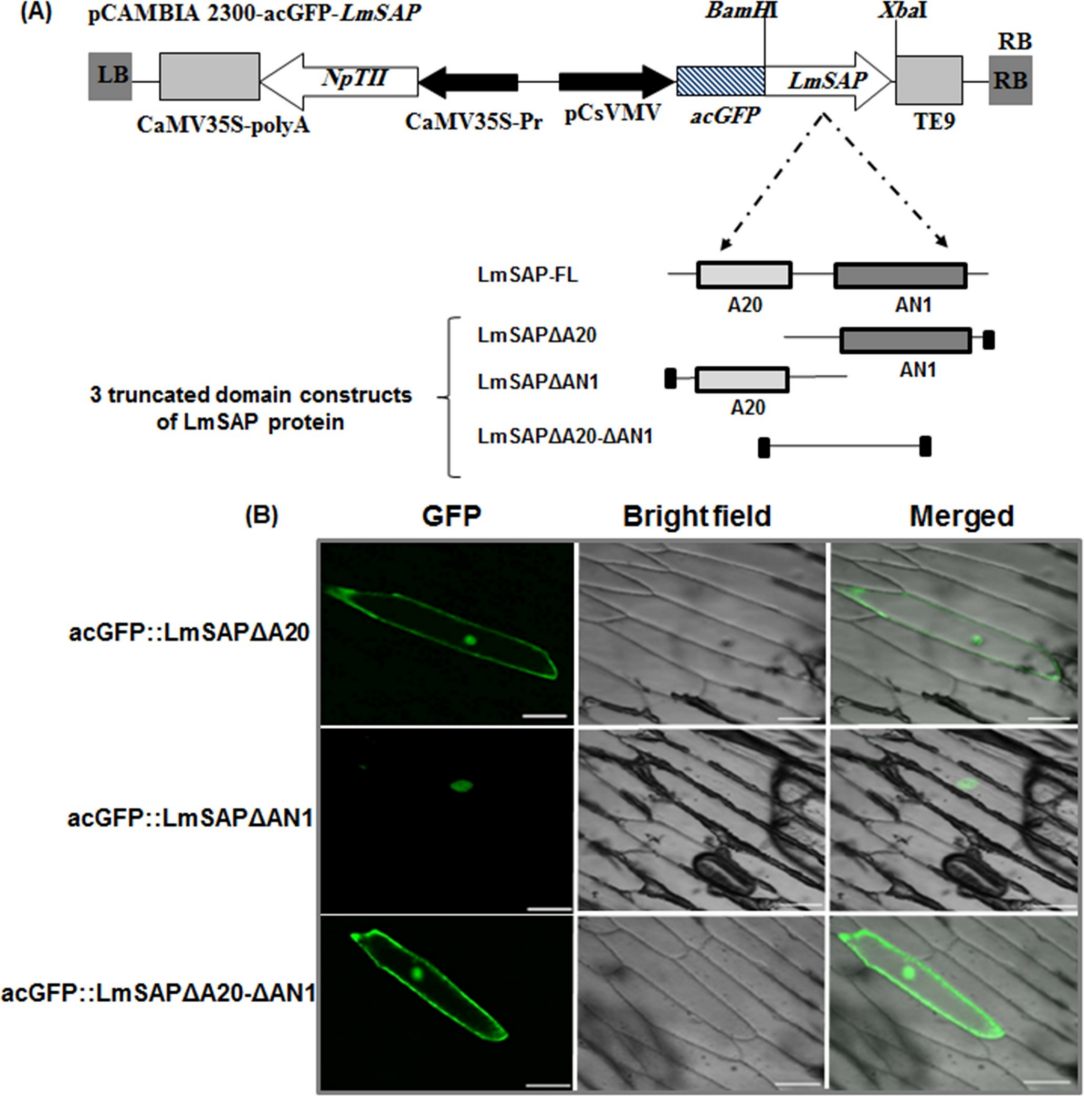
Subcellular localization of the truncated forms of LmSAP protein. (**A**) Schematic diagram of the pCAMBIA2300-acGFP-LmSAP constructs showing the three truncated domains, LmSAPΔA20, LmSAPΔAN1, and LmSAPΔA20-ΔAN1. (**B**) Localization of LmSAPΔA20, LmSAPΔAN1, and LmSAPΔA20-ΔAN1 constructs with the acGFP fusion protein in onion epidermal cells following particle bombardment, as visualized using confocal microscopy. GFP (left), bright field (middle), and merged (right) images are shown. Bars = 100 μm.

Previous study reported that the LmSAP::GFP fusion was exclusively localized in the nucleus [29]. As LmSAP protein contains A20 and AN1 domains, we wanted to find out which region of the protein is responsible for these localization patterns. Three LmSAP-truncated domains were fused in-frame to the C-terminus of the acGFP coding sequence: acGFP::*LmSAPΔA20*, acGFP::*LmSAPΔAN1* and acGFP::*LmSAPΔA20-ΔAN1* were made (Fig 3A). Transient expression in onion epidermal cells showed exclusive localization of acGFP::LmSAPΔAN1 with A20 domain in nucleus, whereas acGFP::*LmSAPΔA20* and acGFP::*LmSAPΔA20-ΔAN1* constructs were localizing in nucleo-cytoplasm (Fig 3B).

### In silico tertiary structure modeling and comparison of LmSAP-truncated domains

The three-dimensional structures of native LmSAP and its truncated forms were analyzed to understand the changes in the locations and functions of the LmSAP-truncated forms. Deletion of the A20 and AN1 domains in the truncated forms of LmSAP led to their structural differences. The models of LmSAPΔA20, LmSAPΔAN1, and LmSAPΔA20-ΔAN1 were compared using structural superposition and RMSD analysis to demonstrate the structural changes caused by the deletion of the domains (S1A–S1D Fig). The superimposition of the predicted models of LmSAPΔAN1, LmSAPΔA20, and LmSAPΔA20-ΔAN1 with that of native LmSAP (LmSAP-FL) showed RMSD values of 20 Å, 23.8 Å and 23.02 Å, respectively (S1E–S1G Fig). These RMSD values indicate that all the predicted models of the truncated forms of LmSAP exhibit structural differences compared to the predicted model of native LmSAP. However, LmSAP-AN1 model presented fewer structural differences compared to the LmSAP-FL model (RMSD = 20 Å) than the other predicted models.

Structural comparison of the predicted three-dimensional models of LmSAP-ΔAN1, LmSAP-ΔA20, and LmSAPΔA20-ΔAN1 against that of LmSAP-FL showed that the deletion of the A20 and AN1 domains of LmSAP lead to enormous changes in its structure (S1A–S1G Fig). In case of the LmSAP truncated form with the AN1 domain deleted, although the structure of the protein is vastly different from that of the native LmSAP-FL protein, the A20 domain is enough to maintain the biological function and nuclear localization of the native LmSAP protein. However, the dramatic structural changes in the LmSAP protein caused by the deletion of the A20 domain lead to the loss of its function and localization. These findings suggest that the presence of the A20 domain is vital for maintaining the structural and functional stability of LmSAP.

### Overexpression of *LmSAP* and *LmSAP*-truncated domains up-regulates expression of GA biosynthesis-related genes and increases GAs level in transgenic tobacco plants

Since tobacco plants that overexpressed *LmSAPΔA20* and *LmSAPΔA20-ΔAN1* truncated *LmSAP* showed significant dwarfism under abiotic stress conditions (Fig 1), we predicted that *LmSAP* may regulate the expression of genes encoding key enzymes involved in GA biosynthesis and subsequently influences the endogenous GAs level.

**Fig 4:**
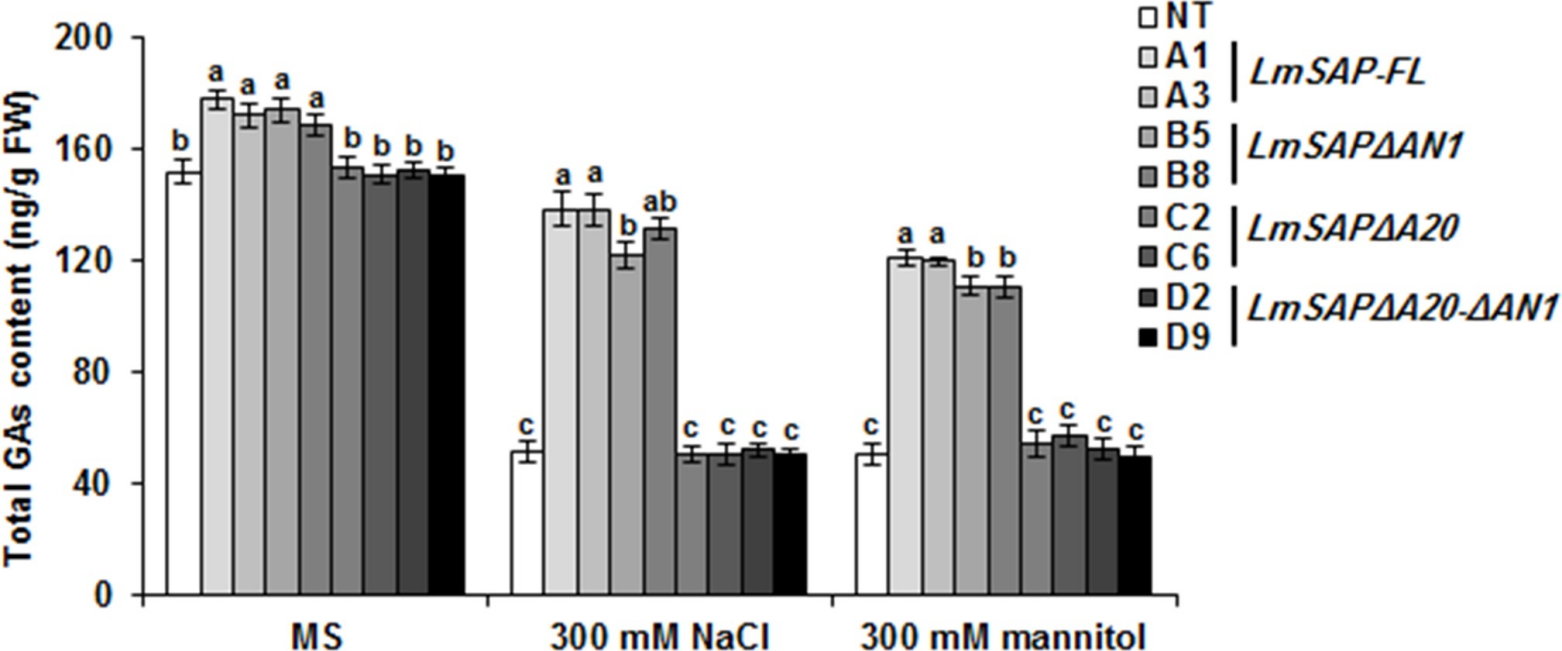
Endogenous GAs content quantified using HPLC in *LmSAP*-overexpressing lines and non-transgenic (NT) plants grown on MS medium (control), MS medium supplemented with 300 mM NaCl_,_ and MS medium supplemented with 300 mM mannitol. Data represent the means ± SEM of three biological replicates. Means denoted by the same letter did not differ significantly at *p*< 0.05.

Quantification of total endogenous GAs in NT and *LmSAP* transgenic plants under control, salt, and osmotic stress conditions revealed increases of 17% and 11% in total GAs content in transgenic *LmSAP* and *LmSAPΔAN1* overexpressing plants, respectively, compared to the corresponding content in NT, *LmSAPΔA20*, and *LmSAPΔA20-ΔAN1* plants, all of which showed a relatively similar GAs content under control conditions (Fig 4). However, an approximate 22% decrease in endogenous GAs content was observed in *LmSAP* and *LmSAPΔAN1* transgenic plants under salt and osmotic stress conditions compared to the control conditions, in spite of which, total GAs content remained two-fold higher than the corresponding content in NT, *LmSAPΔA20*, and *LmSAPΔA20-ΔAN1* plants under the same salt and osmotic stress conditions (Fig 4). These results suggest that the enhanced tolerance observed was closely related to the role of the LmSAP A20 domain in maintaining GA homeostasis under stressful conditions.

**Fig 5:**
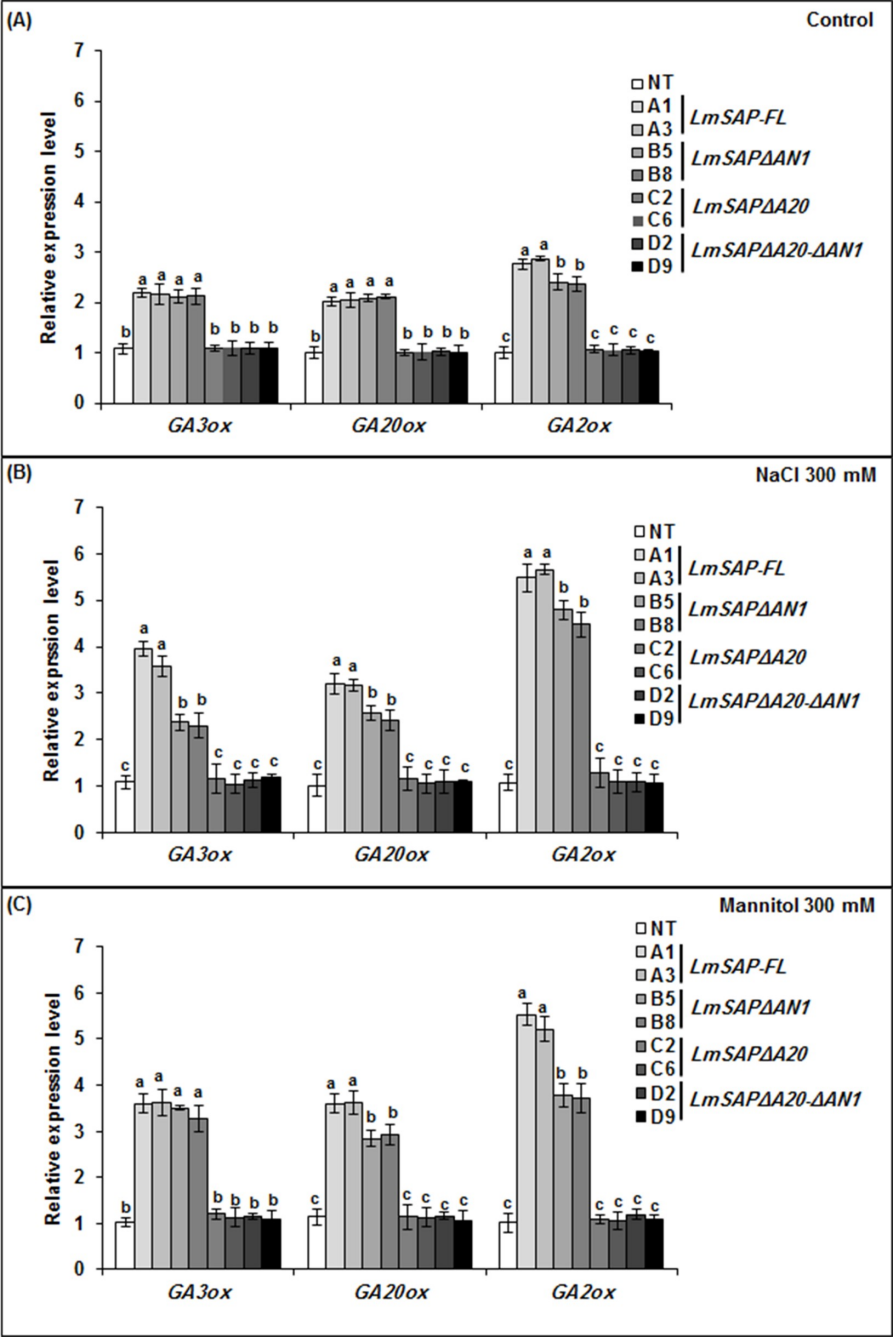
The mRNA level of *GA3ox*, *GA2ox*, and *GA20ox* genes examined by real-time PCR in *LmSAP*-overexpressing lines and non-transgenic (NT) plants grown on (**A**) MS medium (control), (**B**) MS medium supplemented with 300 mM NaCl_,_ or (**C**) MS medium supplemented with 300 mM mannitol. Data represent the means ± SEM of three technical replicates. Means denoted by the same letter did not differ significantly at *p*< 0.05.

RT-qPCR analyses of the three GA-related genes *NtGA3ox*, *NtGA20ox*, and *NtGA2ox*, which encode GA3b-hydroxylase, GA20-oxidases, and GA2-oxidase, respectively, showed increased expression in *LmSAP* and *LmSAPΔAN1* expressing lines as well as relatively similar expression in NT and transgenic plants grown under control conditions (Fig 5A). In contrast, under both salt and osmotic stress conditions, the expression levels of these three GA metabolism genes were significantly up-regulated in *LmSAP* and *LmSAPΔAN1* transgenic tobacco plants as compared with NT or *LmSAPΔA20*, and *LmSAPΔA20-ΔAN1* transgenics (Fig 5B and 5C). However, the magnitude of the increase in the expression levels of these genes in *LmSAPΔAN1* transgenic tobacco plants was reduced relative to *LmSAP* transgenic tobacco plants. Increased expression of *NtGA20ox*, *NtGA3ox*, and *NtGA2ox* in tobacco plants overexpressing *LmSAP* and *LmSAPΔAN1* conferred enhanced tolerance to salt or osmotic stress as compared with NT plants, as well as *LmSAPΔA20* and *LmSAPΔA20-ΔAN1* transgenic plants. These results suggest that the LmSAP A20 domain plays an important role in GA-mediated growth regulation.

### Rescue of dwarf phenotypes in *LmSAPΔA20* and *LmSAPΔA20-ΔAN1*-overexpressing tobacco lines under abiotic stress conditions by application of exogenous GA_3_

**Fig 6:**
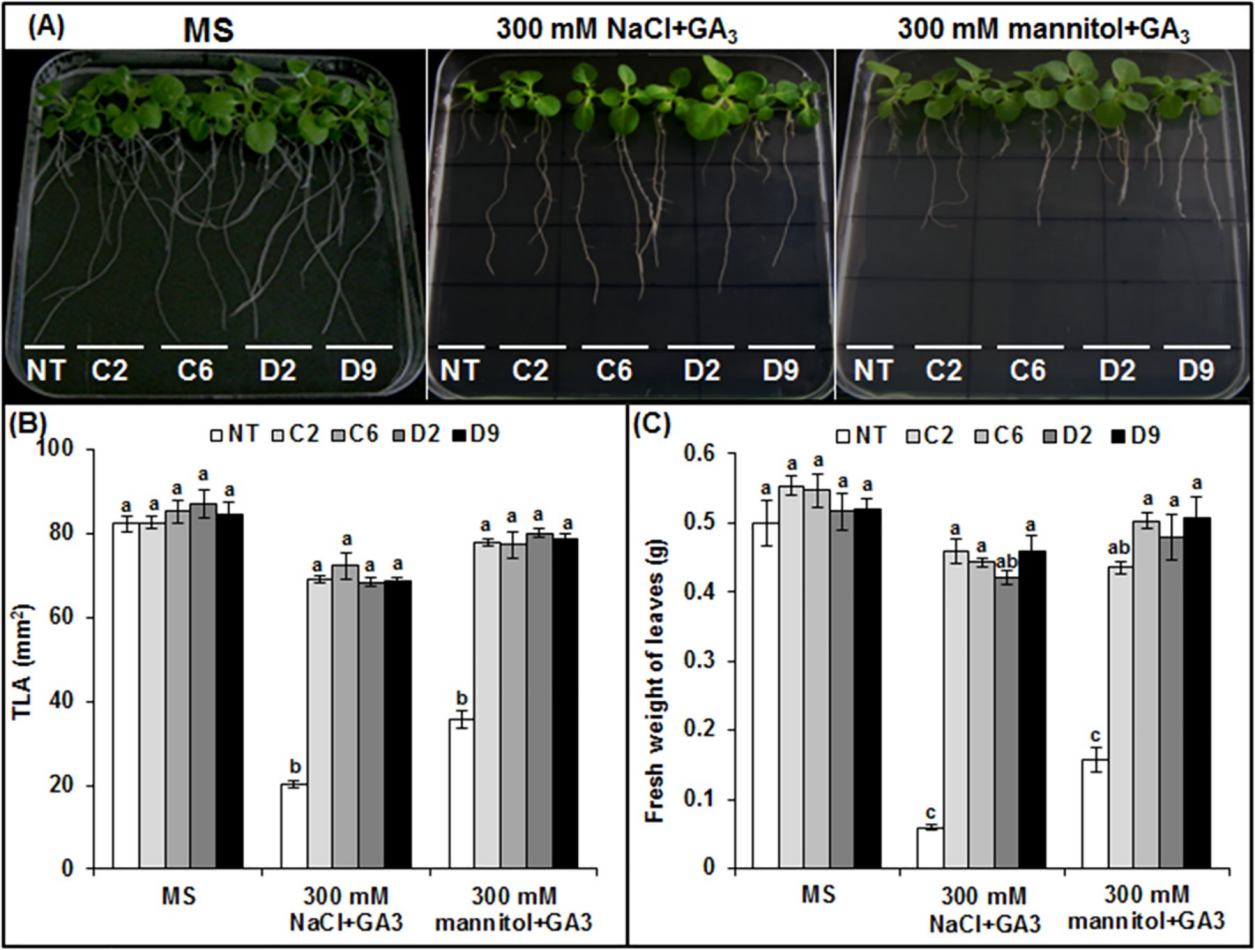
Response of transgenic tobacco plants overexpressing the *LmSAP* gene to exogenous GA_3_. (**A**) Phenotypes of non-transgenic (NT) and transgenic lines (C2, C6, D2, and D9) 2 weeks after *in vitro* incubation on MS medium (control), on MS medium containing 300 mM NaCl and 100 μM GA_3,_ or on MS medium containing 300 mM mannitol and 100 μM GA_3_. (**B**) Total leaf area (TLA) and (**C**) fresh weight of leaves of NT and transgenic plants (C2, C6, D2, and D9) grown on MS medium (control), MS medium containing 300 mM NaCl and 100 μM GA_3_, or MS medium containing 300 mM mannitol and 100 μM GA_3_. Data represent the means ± SEM. Means denoted by the same letter were not significantly different at p < 0.05.

The overexpression of *LmSAPΔA20* and *LmSAPΔA20-ΔAN1* results in dwarfism and smaller leaves in tobacco plants exposed to salt or osmotic stress under *in vitro* conditions [19]. To test whether exogenous GA_3_ treatment could rescue this phenotype, NT tobacco plants and two homozygous transgenic plants from *LmSAPΔA20* and *LmSAPΔA20-ΔAN1* constructs (labeled C2, C6, D2, and D9) were treated with 100 μM GA_3_ on MS solid medium supplemented with 300 mM NaCl or 300 mM mannitol. Two weeks after beginning the salt or osmotic stress treatments with GA_3_, the dwarf phenotype of C2, C6, D2, and D9 tobacco lines was fully restored with the expansion of stem and leaves compared to the phenotype of NT plants after GA_3_ treatment (Fig 6A). No differences in growth were observed between (C2, C6, D2, and D9) transgenic and NT plants under control conditions. The total leaf area (TLA) and biomass production (fresh weight of leaves) were also investigated during growth under control (MS) and stress conditions (salt or osmotic) in NT and transgenic (C2, C6, D2, and D9) plants (Fig 6B and 6C). In the absence of stress, similar TLA values were obtained in all transgenic and NT plants (~80 mm^2^). These values decreased, by approximately 25% and 12.5% in the C2, C6, D2, and D9 lines in the presence of 300 mM NaCl or 300 mM mannitol with 100 μm GA_3_, respectively. In contrast, TLA reduction values in NT plants were 75% and 56% under 300 mM NaCl or 300 mM mannitol with 100 μm GA_3_, respectively (Fig 6B). Nevertheless, considerable increases were observed in the fresh weights of leaves in the transgenic C2, C6, D2, and D9 lines compared to NT plants in the presence of 300 mM NaCl or 300 mM mannitol with 100 μm GA_3_ (Fig 6C). Collectively, these results indicate the involvement of GA_3_ in altering the growth of transgenic plants with LmSAP truncated-domains.

## Discussion

Several members of the stress-associated protein (SAP) family, characterized by the presence of A20 and AN1 domains, have important roles in the abiotic stress response [16, 30, 31]. LmSAP, a member of the SAP family, increases tolerance to salt and osmotic stress under *in vitro* conditions primarily, via its A20 domain. The deletion of the A20 domain does not affect the morphology of transgenic plants under normal conditions; however, it does negatively affect stem elongation and leaf development in transgenic plants under salt and osmotic stress conditions [19]. Herein, we sought to demonstrate the involvement of the A20 and AN1 domains of LmSAP in GA homeostasis in tobacco plants subjected to abiotic stress.

Salt and drought stress tolerance essays in greenhouses showed that the transgenic lines that overexpress *LmSAP-FL* and *LmSAPΔAN1* were more efficient in terms of development (plant height and weight) and seed production compared to NT plants and transgenic lines that overexpress *LmSAPΔA20* and *LmSAPΔA20-ΔAN1*. These results support those of previous study under *in vitro* conditions reported by Ben Saad et al. [19]. Furthermore, these findings reveal that the A20 domain is crucial for LmSAP to improve the tolerance of transgenic tobacco plants to salt and drought stresses in greenhouse conditions. However, the morphological responses of the C2 and D2 transgenic lines which express *LmSAPΔA20* and *LmSAPΔA20-ΔAN1*, respectively, to salt and drought stresses were similar to the phenotype of GA-deficient mutant plants, that were dwarf due to defects in stem elongation. Thus, dwarfism is a common phenotype for several GA-synthesis enzymes defective mutants [9].

Following the application of exogenous GA_3_ at different concentrations, the expression of *LmSAP* was gradually induced in *Lobularia maritima*. The maximum level of *LmSAP* was reached 6 h after the application of 50 μM GA3. Then, the level of *LmSAP* returned to the levels of the control condition. This suggests a regulatory role for LmSAP in GA_3_ homeostasis. Similar results were recently reported which confirm the involvement of two rice A20/AN1-type zinc-finger proteins, ZFP185 and OsDOG, in GAs biosynthesis and alteration of phyto-hormonal homeostasis [17, 18]. However, overexpression of *ZFP185* and *OsDOG* increases the sensitivity of transgenic rice plants to abiotic stresses due to the negative modulation of GA levels [17, 18].

GA is an essential phytohormone mediating shoot elongation, flowering, and nutrient mobilization, and thus enables plants to adapt to unfavorable environmental conditions [32–34]. In particular, exposure to abiotic stresses such as salt, osmotic, and cold stresses downregulates GA synthesis in plants, thereby restricting their growth [15]. GA20- and GA3-oxidases are key enzymes in the pathway generating bioactive GAs, whereas GA2-oxidase is critical to maintaining GA homeostasis [9, 35]. Herein, compared with NT plants and the *LmSAPΔA20* and *LmSAPΔA20-ΔAN1*-expressing lines, we found upregulated expression of GA metabolism-related genes (*GA2ox*, *GA20ox*, and *GA3ox*) and subsequently quantified higher total endogenous GAs content in *LmSAPΔAN1*- and *LmSAP*-overexpressing lines under salt and osmotic stress conditions. Furthermore, only the LmSAPΔAN1truncated form, with an intact A20 domain, retained the nuclear localization typical of LmSAP harboring both the A20 and AN1 domains [29]. By contrast, the truncated forms, LmSAPΔA20 and LmSAPΔA20-ΔAN1, showed a nucleo-cytoplasmic localization. These results suggest that the A20 domain on the LmSAPΔAN1-truncated is essential for maintaining structural stability and nuclear localization in addition to its possible transcriptional activator function. Collectively, these results suggest that the dwarfing phenotype of the *LmSAPΔA20* and *LmSAPΔA20-ΔAN1*-overexpressing lines observed under abiotic stress conditions might be due to lack of GA homeostasis regulation. Indeed, overexpression of *GA20ox* genes has been shown to induce elongated phenotypes in transgenic lines of numerous plant species. For instance, Huang et al. [36] reported that transgenic *Arabidopsis* lines overexpressing *AtGA20ox* exhibited a GA-overproducing phenotype characterized by a longer hypocotyl, accelerated stem elongation, and early flowering. Similarly, in B142 rice mutant strain, *OsGA20ox1* upregulation induced an elongated phenotype with internode overgrowth. In contrast, loss of *GA20ox-2* function caused a semi-dwarf phenotype in IR8 rice. Jia et al [37] reported reduced expression of the *HvGA20ox-2* associated with semi-dwarfing in Baudin barley variety. However, transgenic tobacco lines overexpressing *GA3ox* from pea did not exhibit apparent morphological changes [38]. Nonetheless, *GA2ox* genes are involved in feedback regulation in the GA pathway. Most studies on overexpression of *GA2ox* genes from rice, *Arabidopsis*, tobacco, and *Jatropha* indicate a dwarf phenotype in transgenic plants, owing to the negative regulation of GA content [39–43]. Thus, the dwarf phenotype of *LmSAPΔA20* and *LmSAPΔA20-ΔAN1*-overexpressing lines observed under abiotic stress conditions in this study might be attributed to the lack of GA homeostasis regulation. Application of exogenous GA3 under stress conditions rescued this dwarf phenotype. Moreover, the normal phenotype could be restored in GA-deficient dwarfs by application of exogenous GA, indicating that the mutations usually cause deficiency in the GA metabolic pathway. This suggests that LmSAP, mainly with the A20 domain, is involved in the upregulation of three GA metabolism-related genes (*GA2ox*, *GA20ox*, and *GA3ox*), and thus regulates GA homeostasis under abiotic stress conditions. LmSAP might thus function through DNA-protein or protein-protein interactions, mainly via the A20 zinc-finger domain, to regulate GA metabolism-related genes.

In conclusion, we have demonstrated that LmSAP, encoded by an A20/AN1 SAP gene, is involved in maintaining GA homeostasis under abiotic stress conditions by regulating the expression of GA metabolism-related genes in transgenic tobacco, mainly via its A20 domain. Further research using transcriptomic (RNA-Seq) and proteomic (two-hybrid system) approaches are needed to elucidate the molecular mechanism of LmSAP in regulating GA homeostasis.

## Supporting information

S1 Fig Prediction and superimposition of the tertiary structures of the native and truncated forms of LmSAP. Predicted tertiary structures of LmSAP-FL (**A**), LmSAPΔAN1 (**B**), LmSAPΔA20 (**C**), and LmSAPΔA20-ΔAN1 (**D**). Superimposition of the predicted tertiary structures of LmSAPΔAN1 (**E**), LmSAPΔA20 (**F**), and LmSAPΔA20-ΔAN1 (**G**) on the predicted model of the native LmSAP (red). Root mean square deviation (RMSD) values are indicated within the brackets.
(TIF)

S1 Table Sequences of primers used in RT-qPCR analysis. (DOC)
