# Supplementary figures and images for: A Lobularia maritima LmSAP protein modulates gibberellic acid homeostasis via its A20 domain under abiotic stress conditions
Source: PLoS One. 2020 May 19;15(5):e0233420. doi: 10.1371/journal.pone.0233420 (PMC7237032; doi:10.1371/journal.pone.0233420)

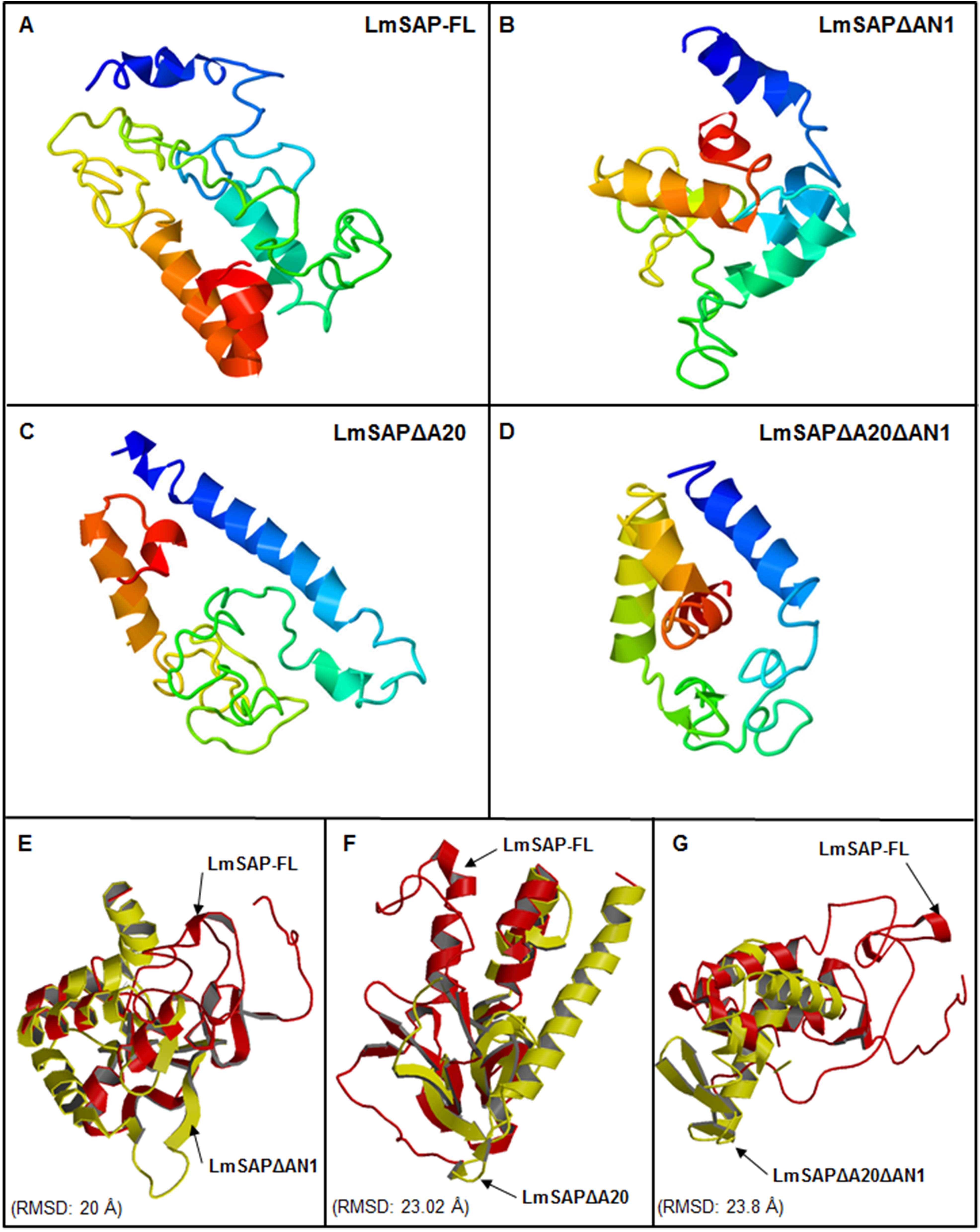

Supplement: S1 Fig — Predicted tertiary structures of LmSAP-FL (A), LmSAPΔAN1 (B), LmSAPΔA20 (C), and LmSAPΔA20-ΔAN1 (D). Superimposition of the predicted tertiary structures of LmSAPΔAN1 (E), LmSAPΔA20 (F), and LmSAPΔA20-ΔAN1 (G) on the predicted model of the native LmSAP (red). Root mean square deviation (RMSD) values are indicated within the brackets. (TIF) [file pone.0233420.s001.tif]
