# Supplementary material for: A Lobularia maritima LmSAP protein modulates gibberellic acid homeostasis via its A20 domain under abiotic stress conditions
Source: PLoS One. 2020 May 19;15(5):e0233420. doi: 10.1371/journal.pone.0233420 (PMC7237032; doi:10.1371/journal.pone.0233420)
Supplement: S1 Table — (DOC) [file pone.0233420.s002.doc]

**S1 Table**. Sequences of primers used in RT-qPCR analysis.

| **Primers** | **Sequences（5' → 3'）** |
| --- | --- |
| **LmSAP-F** | TTAGAATTCATGGCTCAGAGTTCGGAG |
| **LmSAP-R** | TTAGAATTCTTAATCGAAAAAACTTTGA |
| **qDEF-F** | GCGCTTCTTTGCAACTGTGTTACTTATAGC |
| **qDEF-R** | TCAGACAAACGGTGGCACAGTTG |
| **qPR1-F** | TGGGATTTGTTCTCTTTTCACA |
| **qPR1-R** | TTACGCCAAACCACCTGAGT |
| **qPR2-F** | ATCCTTCAAGAGCCAATGGA |
| **qPR2-R** | TCCAAAAGGGCATCAAAAAG |
| **qVSP2-F** | TCCATCAACTACGCCAACTG |
| **qVSP2-R** | CGGTTTTGGAGTCGTATTGG |
| **qLOX3-F** | AGGAGATGCGGAGATTGTTG |
| **qLOX3-R** | CAGGATCAGCGTTCCTTTTC |
| **qACT-F** | GTGCCCATTTACGAACGATA |
| **qACT-R** | GAAGACTCCATGCCGATCAT |
